# The fetal glucose steal: an underappreciated phenomenon in diabetic pregnancy

**DOI:** 10.1007/s00125-016-3931-6

**Published:** 2016-03-19

**Authors:** Gernot Desoye, Christopher J. Nolan

**Affiliations:** Department of Obstetrics and Gynaecology, Medical University of Graz, Auenbruggerplatz 14, 8036 Graz, Austria; Department of Endocrinology, Canberra Hospital, Canberra, ACT Australia; Department of Endocrinology, Australian National University Medical School, Canberra, ACT Australia

**Keywords:** Diabetic fetopathy, Fetal glucose steal, Fetal hyperinsulinaemia, Gestational diabetes mellitus, Placenta, Pregnancy, Review, Transplacental glucose transfer, Type 1 diabetes, Type 2 diabetes

## Abstract

Adverse neonatal outcomes continue to be high for mothers with type 1 and type 2 diabetes, and are far from eliminated in mothers with gestational diabetes mellitus. This is often despite seemingly satisfactory glycaemic control in the latter half of pregnancy. Here we argue that this could be a consequence of the early establishment of fetal hyperinsulinaemia, a driver that exaggerates the fetal glucose steal. Essentially, fetal hyperinsulinaemia, through its effect on lowering fetal glycaemia, will increase the glucose concentration gradient across the placenta and consequently the glucose flux to the fetus. While the steepness of this gradient and glucose flux will be greatest at times when maternal hyperglycaemia and fetal hyperinsulinaemia coexist, fetal hyperinsulinaemia will favour a persistently high glucose flux even at times when maternal blood glucose is normal. The obvious implication is that glycaemic control needs to be optimised very early in pregnancy to prevent the establishment of fetal hyperinsulinaemia, further supporting the need for pre-pregnancy planning and early establishment of maternal glycaemic control. An exaggerated glucose steal by a hyperinsulinaemic fetus could also attenuate maternal glucose levels during an OGTT, providing an explanation for why some mothers with fetuses with all the characteristics of diabetic fetopathy have ‘normal’ glucose tolerance.

## Introduction

Despite significant advances in glycaemic control in the management of diabetes in pregnancy, adverse outcomes for the fetus are still very common, particularly for pre-existing type 1 and type 2 diabetes. For example, in a recent review of 12 studies comprising 14,099 type 1 diabetic pregnancies the RRs conferred by diabetes over the background population for perinatal mortality, congenital malformations and preterm birth were all significantly increased (3.7, 2.4 and 4.2, respectively) [1]. Of particular note, 54.2% of neonates of type 1 diabetic pregnancies were born large for gestational age (LGA) compared with 10.0% in the background population— RR of 4.5 [1]. In addition, in gestational diabetes mellitus (GDM), standard approaches to treatment, while reducing the rates of LGA, do not fully normalise neonatal outcomes, including the rates of neonatal hypoglycaemia, raised cord blood C-peptide levels and neonatal fat mass [2–4].

In this article we propose that an exaggerated fetal glucose steal phenomenon, driven by fetal hyperinsulinaemia, contributes to diabetic fetopathy, including excessive fetal fat accretion. After introducing the glucose steal phenomenon, we review what is known about the early development of insulin secretion in the fetus. We then discuss the need for tight glucose control in the first and early second trimesters to prevent fetal hyperinsulinaemia and exaggeration of the glucose steal with its implications for the fetus.

## The principle of the fetal glucose steal phenomenon in normal and diabetic pregnancies

In considering determinants of glucose transfer from mother to fetus, we focus mostly on those of the mother rather than those of the fetus. Maternal glucose metabolism adapts to pregnancy so as to provide a continuous supply of glucose to the fetus. These hormone-regulated adaptations include increased hepatic glucose production in the fasting state, to ensure a glucose supply at all times, and maternal peripheral insulin resistance, which spares glucose for the fetus [5].

Mentioned rarely is the role of the fetus (and placenta) and its propensity to act as a glucose sink, stealing glucose from the mother. This process has been termed the ‘fetoplacental glucose steal phenomenon’ [6]. It was originally established in sheep [7, 8] and elaborated in detail in the rat [6]. There is also indirect evidence that it operates in humans [9, 10].

**Fig. 1:**
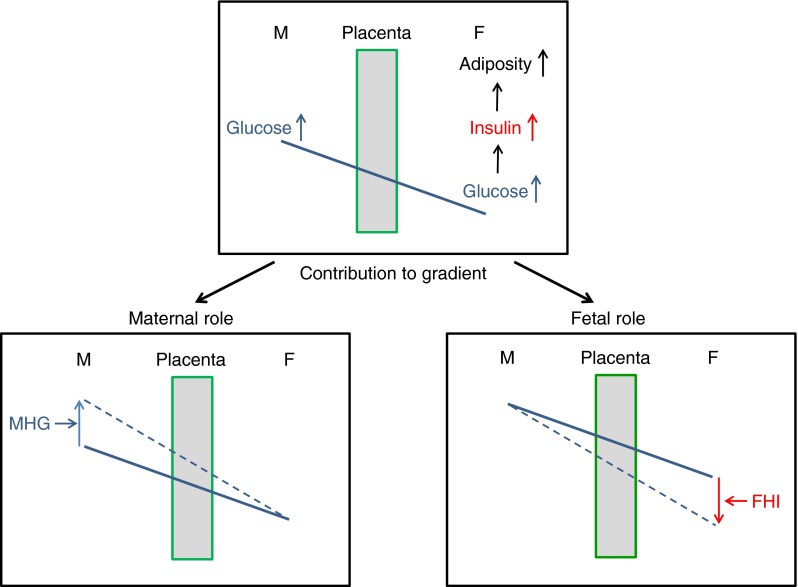
Maternal (M) glucose is transferred to the fetus (F) across the placenta down a concentration gradient. The glucose gradient is determined by both maternal and fetal glucose levels. Maternal high glucose (MHG) and/or lower fetal glucose will steepen the gradient, leading to augmented glucose flux into the fetus. MHG is a consequence of inadequate control of maternal diabetes, which promotes increased glucose transfer to the fetus, increased fetal blood glucose, increased stimulation of insulin release from fetal islets and, consequently, fetal hyperinsulinaemia (FHI), as described by Pedersen and Osler [12]. Importantly, FHI, by increasing the rate of glucose utilisation by the fetus, will lower fetal glucose, thereby increasing the steepness of the transplacental glucose gradient and the rate of glucose transfer. In effect, MHG pushes glucose and FHI pulls glucose (the fetal glucose steal) across the placenta to the fetus. The ensuing increased glucose delivery from diabetic mothers, together with the FHI, stimulates fetal triacylglycerol formation and the deposition of excess fetal adipose tissue

All available evidence suggests that the placenta is a passive conduit for the proportion of maternal glucose destined to reach the fetus, at least at the end of gestation [11]. Thus, the maternal-to-fetal glucose flux is mostly dictated by the maternal-to-fetal glucose concentration gradient, i.e. the glucose concentration difference between the maternal and fetal compartments. Therefore, any concentration change in either compartment will modify the concentration gradient and affect glucose flux (Fig. 1).

Maternal glucose concentrations are determined not only by maternal factors such as the type of diabetes and how well it is managed, but also by the rate of glucose uptake in the fetus. Fetal glucose concentrations are determined by both glucose appearance, by transplacental glucose transfer from the mother, and glucose disappearance, by its uptake into fetal tissues, the latter being influenced by fetal insulin levels, fetal insulin sensitivity and fetal weight. Thus, factors on either side of the placenta determining maternal and fetal glucose concentrations are determinants of the transplacental glucose gradient (Fig. 1).

We propose that the fetal glucose steal phenomenon is exaggerated in diabetic pregnancies as a consequence of fetal hyperinsulinaemia and, in addition to maternal factors, this steal contributes to diabetic fetopathy. The hallmark of diabetic fetopathy is fetal hyperinsulinaemia, which drives fetal fat accumulation, very often resulting in an LGA birth. However, increased adiposity can also occur in infants of diabetic women who have normal birthweight [4].

Because of its dual role as a growth factor and anabolic hormone, fetal insulin has long been recognised as the major driver of excess adipose tissue deposition in fetuses of mothers with diabetes [12, 13]. Diabetic pregnancies are associated with elevated fetal insulin levels due to increased maternal glucose reaching the fetal circulation, which stimulates insulin release from the fetal pancreas (Pedersen hypothesis, Fig. 1). However, other insulin secretagogues such as amino acids and, potentially, fatty acids may also contribute [14, 15].

In newborns of diabetic mothers, glucose clearance into tissues is much faster than in newborns from normal pregnancies, often causing neonatal hypoglycaemia due to neonatal hyperinsulinaemia [16]. Hyperinsulinaemia in the fetus is likely also to accelerate glucose clearance into fetal tissues and increase the fetal glucose steal. Thus, the maternal–fetal glucose gradient can be steepened in diabetic pregnancy by both maternal (poor glycaemic control) and fetal (hyperinsulinaemia) factors, resulting in increased glucose transfer to the fetus. Furthermore, even in the presence of normal maternal glucose levels, fetal hyperinsulinaemia will still lower fetal glucose concentrations, sustaining a high glucose gradient and an exaggerated glucose steal (Fig. 1).

### Early development of fetal hyperinsulinaemia

The human pancreas begins to develop 4 weeks after conception, and first insulin deposits can be found between weeks 7 and 8 [17]. In vitro fetal pancreases release insulin as early as 11 weeks of gestation [18]. Amniotic fluid insulin (AFI), which originates from loss into fetal urine, is just detectable at 12 weeks [19] and more easily measurable from 14 weeks onwards [20]. AFI can be used as a surrogate for fetal serum insulin, as AFI measured at 31 weeks has been shown to be highly correlated with cord blood insulin measured at birth (*r* = 0.853; *p* = 0.0001) [9].

While neonatal hyperinsulinaemia as a consequence of maternal diabetes is well established to occur as determined by the measurement of cord blood insulin or C-peptide from samples collected immediately after delivery [21], its onset in pregnancy is less well understood. It has long been thought that the fetal pancreas is unresponsive to glucose changes early in pregnancy [22]. While this appears to hold true for normal pregnancies, the fetal pancreas of pregnant women with diabetes is already sensitive to glucose in vitro at 12 weeks [18]. This explains why AFI levels can be elevated before the diagnosis of GDM [20, 23]. Elevated AFI concentrations, beginning at 14 weeks, are associated not only with the mother’s risk of developing GDM [20, 23] but also with the risk of the fetus having a birthweight >90th centile [20].

The cause of early fetal hyperinsulinaemia is not fully understood; however, it is highly correlated with maternal glycaemic control early in pregnancy, such that early maternal hyperglycaemia in type 1 and type 2 diabetic pregnancies, and most probably GDM, is likely to be the major driver. Roles for alterations in other maternal nutrient levels associated with by diabetes, however, may also be important. The maternal diabetic state not only leads to beta cell hyperplasia in the fetus [24, 25] but also seems to accelerate the maturation of the beta cell stimulus–secretion coupling mechanism [26]. It is of note that fat deposition in the fetus begins at around 14 weeks, which is the time of onset of early fetal hyperinsulinaemia [27].

## The importance of maternal metabolism in the first trimester of pregnancy

The pathophysiological relationship between maternal glucose and fetal neonatal adiposity mediated through fetal insulin levels was proposed more than six decades ago [12] and has recently been confirmed in a large cohort of non-diabetic women of different ethnicities [28]. This raises the question about when in pregnancy the metabolic perturbations in the mother begin to influence the fetus. While this large cohort study established that it occurs at 24–28 weeks’ gestation [28], there is evidence that earlier periods may play an important role. Although body composition was not measured and, hence, body fat was not an outcome, a continuous relationship between maternal fasting glucose measured as early as 9–10 weeks’ gestation and the risk of an LGA birth has been shown [29]. Likewise, random blood glucose in the first trimester was positively correlated with proinsulin levels in the cord blood [30]. The influence of early pregnancy glycaemia is also seen in the reports of stronger associations of maternal first and second trimester glycosylated haemoglobin levels, compared with later measurements, with birthweight and occurrence of LGA [1, 31–33]. Consistent also is the finding that excessive fetal growth in pre-gestational diabetes is evident by serial ultrasound in many pregnancies from 18 to 24 weeks and that those with established LGA prior to 30 weeks are most likely to result in greater severity macrosomia at birth [31, 34, 35].

The period when the metabolic environment has an effect on fetal growth may even extend to pre-pregnancy. The influence of pre-pregnancy metabolic changes on fetal development may be mediated through modification of oocyte metabolism [36], predominantly of their mitochondria [37], through changing early embryonic growth and later growth trajectories.

## Clinical consequences of the fetal glucose steal

### Glucose steal and associations of early and late pregnancy glycaemic control with diabetic fetopathy

As mentioned above, stronger associations between early rather than late pregnancy glycosylated haemoglobin levels with LGA are often reported [1, 31–33]. This is not, however, a universal finding of all studies, as some show a greater effect of third trimester glycaemic control [38, 39]. Overall, these studies emphasise the importance of optimal glycaemic control through the whole of pregnancy to prevent diabetic fetopathy.

Poor glycaemic control early in pregnancy will result in the establishment of fetal hyperinsulinaemia, causing an exaggerated fetal glucose steal. As a consequence, the overactive glucose steal will increase the disposal of maternal glucose into the fetus, thus attenuating the levels of maternal hyperglycaemia. Importantly, this effect of lowering maternal glucose driven by the fetus will be greatest in pregnancies with the most hyperinsulinaemic fetuses.

Thus, the challenge for many clinicians working in the field of diabetes in pregnancy that seemingly tight late pregnancy glycaemic control often fails to prevent diabetic fetopathy, at least in type 1 and type 2 diabetic pregnancies, can be partly explained by the fetal glucose steal. This is because fetal hyperinsulinaemia will sustain an increased glucose gradient across the placenta and the glucose steal, even at times of near-normal maternal glycaemia.

Once fetal hyperinsulinaemia is established, however, continued maternal hyperglycaemia into the third trimester will likely compound the effects of the fetal hyperinsulinaemia and glucose steal on accelerated fetal growth, as the maternal–fetal glucose gradient and glucose transfer to the fetus will be greater. This is consistent with reports that show an effect of late pregnancy glycaemia on the adverse outcome of LGA [38, 39].

### Exaggerated glucose steal due to fetal hyperinsulinaemia: risk of masking GDM diagnosis by OGTT

**Fig. 2:**
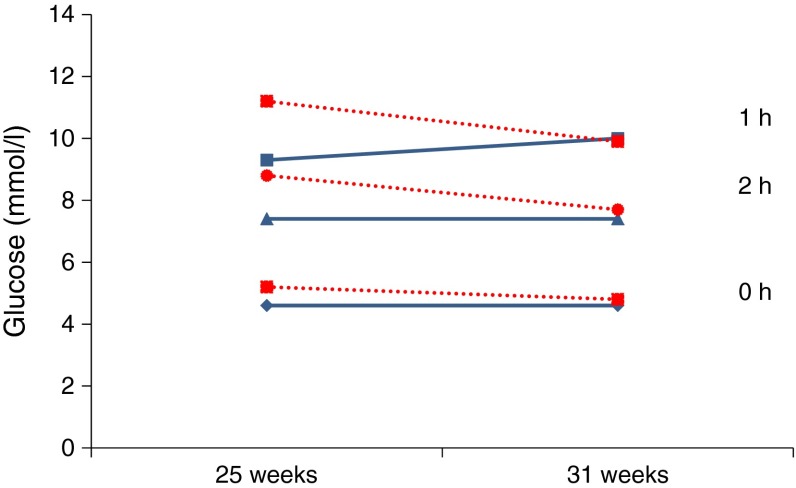
OGTTs were performed in 34 mothers with GDM at 25 and 31 weeks prior to any commencing insulin therapy. Amniocentesis was also performed at week 31 and AFI levels were measured [9]. In the mothers of the 13 normoinsulinaemic fetuses (AFI <48.6 pmol/l; solid blue line), maternal glucose tolerance deteriorated from the first to the second time point as manifested by a higher 1 h post-load glucose value (9.3 vs 10.0 mmol/l; *p* = 0.0006). In the mothers of the 21 hyperinsulinaemic fetuses (AFI >48.6 pmol/l; dotted red line), the glucose levels at 1 and 2 h of the repeated OGTT were lower (significant for the 1 h reading only) than at 25 weeks (1 h, 11.2 vs 9.9 mmol/l, *p* = 0.002; 2 h, 8.8 vs 7.7 mmol/l, *p* = 0.158) [9]. Figure created from data from Weiss et al [9]

Weiss et al assessed changes in the OGTT from 25 to 31 weeks’ gestation in women with GDM categorised according to the presence or absence of fetal hyperinsulinaemia [9]. In women with GDM without evidence of fetal hyperinsulinaemia, determined by AFI <48.6 pmol/l, glucose tolerance deteriorated from 25 to 31 weeks’ gestation (Fig. 2). By contrast, in GDM women with fetal hyperinsulinaemia (AFI >48.6 pmol/l), glucose tolerance improved during the same period (Fig. 2). This can be explained by siphoning of the glucose to the fetus in women with hyperinsulinaemic fetuses, due to the effects of an increase in glucose steal activity [9]. The placentas of hyperinsulinaemic, macrosomic fetuses might also have been larger, such that increased placental glucose consumption could also have contributed to the blunting of the OGTT glucose rise observed in the mothers. Thus, there is a risk that GDM will not be diagnosed in women with the most affected fetuses. Consistent with this, seven of 21 (33%), compared with none of 11 (0%), women with AFI >48.6 pmol/l, compared with AFI <48.6 pmol/l, delivered neonates with birthweight >4,000 g [9].

The data of Weiss et al are important for two reasons (Fig. 2) [9]. First, they support the premise that an exaggerated glucose steal phenomenon operates in diabetic pregnancies complicated by fetal hyperinsulinaemia. Second, they provide a reason why some women with fetuses with all the characteristics of diabetic fetopathy are often found to have ‘normal’ glucose tolerance.

### Can tight glycaemic control later in pregnancy reverse fetal hyperinsulinaemia and the fetal glucose steal?

The findings that treatment of GDM from 24 to 28 weeks can significantly reduce the rate of LGA births suggest it is possible to reverse fetal hyperinsulinaemia, at least when hyperglycaemia is mild [2, 3]. However, the rate of neonatal hypoglycaemia needing intravenous glucose therapy was not significantly reduced in the GDM treatment arms [2, 3]. Furthermore, treatment did not significantly reduce the rate of elevated cord C-peptide levels in the study where this was measured [3]. Despite normalisation of birthweight, management of GDM has not been shown to normalise excess fetal adiposity [4]. Thus, fetal hyperinsulinaemia appears to be difficult to normalise with the usual approach to managing mild maternal hyperglycaemia [2, 3].

Interestingly, in a study in which treatment of GDM with insulin was initiated according to the presence or absence of an elevated AFI concentration, the rate of elevated cord blood C-peptide levels in neonates was markedly reduced, suggesting that there is some potential to reverse fetal hyperinsulinaemia by targeting it in GDM pregnancies [40]. In pre-existing diabetes, however, when normalisation of maternal glucose levels is very difficult to achieve, fetal hyperinsulinaemia is likely to be much more difficult to reverse, as potentially even short periods of maternal hyperglycaemia will sustain it. The association between hyperglycaemic spikes in the third trimester with LGA would be consistent with this premise [38].

## Future directions

Of major importance is an improved understanding of the mechanisms causing early fetal hyperinsulinaemia, as this is the driver that accelerates the fetal glucose steal in diabetic pregnancy. While it may be a consequence of maternal glycaemia only, other nutrients such as amino acids and fatty acids and/or hormones/cytokines could also contribute, particularly in the pregnancies of obese mothers in whom glucose tolerance is normal but cord blood C-peptide levels are shown to be elevated at birth [41]. It will be particularly important to determine the factors that render early glucose signalling competence to fetal islets, as this occurs earlier in diabetic pregnancies. It is already known from elegant studies of the glucokinase gene in the fetus that fetal genetic factors can alter the responsiveness of fetal islets to maternal hyperglycaemia [42]. Improved understanding of fetal (including genetic and epigenetic) factors that determine the likelihood of pathological hyperinsulinaemia in the fetus will also be useful. The role of the placenta in fetal nutrient supply early in pregnancy is entirely unknown. In particular, studies should be undertaken to address the question whether the placenta plays a passive, permissive role or whether it actively contributes to determining the amount of nutrients that will be passed on to the fetus in the early periods of gestation.

The discussion above has focused only on the fetal glucose steal. It is highly conceivable that fetal hyperinsulinaemia could also drive a fetal fatty acid and/or an amino acid steal. Furthermore, an increased supply of fatty acids and amino acids could contribute to the maintenance of fetal insulin hypersecretion and fetal hyperinsulinaemia in situations where maternal glucose levels are relatively well controlled. A fetal fatty acid steal is clearly conceivable, at least for those fatty acids which are transferred by diffusion [43]. For other fatty acids and amino acids the more complex transplacental transfer mechanisms may make a steal phenomenon more complicated.

The high likelihood that fetal hyperinsulinaemia can mask an OGTT diagnosis of GDM through the glucose steal phenomenon requires further confirmation and the clinical consequences need thorough consideration. New methodologies to reliably diagnose the presence of fetal hyperinsulinaemia should also be sought, potentially to indicate the degree of aggressiveness required to improve maternal metabolic control.

Randomised controlled clinical trials focusing on optimisation of glycaemic and metabolic control prior to and during the first trimester of pregnancy in disorders of mild hyperglycaemia, or in women who are overweight and obese even with normoglycaemia, are required to determine whether this can prevent fetal hyperinsulinaemia and excess fetal growth.

Finally, to further improve pregnancy outcomes related to fetal hyperinsulinaemia and exaggeration of the fetal glucose steal in pre-existing type 1 and type 2 diabetes, as well as in GDM, it is necessary to optimise metabolic control early in pregnancy. This will necessitate pre-pregnancy planning for women with pre-existing diabetes, as well as for those at increased risk of GDM, and better means to safely normalise glycaemia.

## Abbreviations

AFI: Amniotic fluid insulin
GDM: Gestational diabetes mellitus
LGA: Large for gestational age
